# A case of multisystem inflammatory syndrome in an African adolescent male: case report

**DOI:** 10.11604/pamj.2021.38.174.27910

**Published:** 2021-02-16

**Authors:** Pramodhini Moodley, Jacob Merika Letlhoo Tsitsi, Denasha Lavanya Reddy, Mohith Debising, Claudia Ickinger

**Affiliations:** 1 Faculty of Health Sciences, University of the Witwatersrand, Department of Internal Medicine, Chris Hani Baragwanath Academic Hospital, Soweto, Johannesburg, South Africa

**Keywords:** Multisystem inflammatory syndrome, children, African, SARS-CoV-2, case report

## Abstract

Since late April 2020, a syndrome now termed Multisystem Inflammatory Syndrome in Children (MIS-C) has been seen in children and adolescents in association with COVID-19 infection. The definition of MIS-C involves fever, organ dysfunction and laboratory confirmation of inflammation in the context of laboratory or epidemiological evidence of SARS-CoV-2 infection in a patient under 21 years of age. Notably, cases are now being identified in adults termed Multisystem Inflammatory syndrome in Adults (MIS-A). Few cases have been reported in sub-Saharan Africa. We report a case of a young African male presenting with a maculopapular rash, persistent fever, elevation in inflammatory markers and a sudden, significant deterioration in cardiac function resulting in cardiogenic shock. The patient responded to intravenous steroids, intravenous immunoglobulin and background inotropic support. The recognition of this disease entity proves even more crucial now amidst the ongoing global wave of COVID-19 infection. It is paramount to identify these patients early, leading to prompt treatment avoiding further morbidity and mortality.

## Introduction

The novel coronavirus disease 2019 (COVID-19) or severe acute respiratory syndrome coronavirus 2 (SARS-CoV-2) was first noted in December 2019 in Wuhan, China [1]. On the 11^th^ of March 2020 the virus was declared a pandemic [1]. As of 12 January 2021, there are 89,048,345 confirmed cases globally, with 1,930,25 deaths in total [2]. The first South African case was reported on the 5^th^ of March 2020. The overall number of cases in this country has increased to 1,246,643 as of 12 January 2021, with case numbers on the rise due the second wave of the disease [2]. Since late April 2020, a syndrome now termed Multisystem Inflammatory Syndrome in Children (MIS-C) (also known as Paediatric Inflammatory Multisystem Syndrome (PIMS)) was seen in children and adolescents in association with COVID-19 infection, with cases now being reported in our local South African setting [3]. This was defined by the Centres for Disease Control (CDC) in May 2020 and involves fever, organ dysfunction and laboratory confirmation of inflammation in the context of laboratory or epidemiological evidence of SARS-CoV-2 infection in a patient under 21 years of age. A review of recently published cases has noted similar findings in adults, termed Multisystem Inflammatory Syndrome in adults (MIS-A) [4]. The case definition of MIS-A includes five similar criteria in those over 21 years of age. We describe a case of a young African adolescent male presenting with features of MIS-C, initially thought to be infection or underlying connective tissue disease, which we believe needs to be entertained as an important differential diagnosis in patients presenting similarly during this phase of the pandemic.

## Patient and observation

### Patient information

On the 20^th^ August 2020, a previously healthy, 17-year-old African male was transferred from a local hospital to the medical admission ward at Chris Hani Baragwanath Academic Hospital (CHBAH), Soweto, Johannesburg. The patient presented with a one week history of fever, generalized rash over the body and eyelids, intermittent headache with associated neck pain, sore throat and a dry cough, preceded by a day history of diarrhoea. There was no history of arthralgias, oral or nasal ulcers, or urethral discharge. There was no prior medical admission, no history of surgery and no significant medication or recreational drug use. He is currently a grade ten scholar. There was no known COVID-19 case contact, however recent travel to Kwa-Zulu Natal two weeks prior was noted (Table 1).

**Table 1:** timeline of events from admission

Day (Date)	Intervention
Pre-admission	1 week history of fever, headache and neck pain and sore throat
	Generalized maculopapular, erythematous rash develops over the body three days before admission
1 (Admission 20/08/2020)	Admitted to Chris Hani Baragwanath hospital
	Continued on IV fluids
	Continued on antibiotics ceftriaxone, day 3
2 (21/08/2020)	Started on acyclovir
	Doxycycline added
	Continued on ceftriaxone
	Persistent temperature noted
3 (22/08/2020)	Drop in blood pressure
	Increase in intravenous fluid rate
	Slight improvement in rash
4 (23/08/2020)	Increase in troponin levels noted
	ECHO: global hypokinesia, ejection fraction:10%
	Intravenous immunoglobulin started
	Dobutamine infusion started
	Gentle furosemide infusion started
5 (24/08/2020)	Intravenous methylprednisolone started
	Serology sent for COVID-19 antibody testing
	Furosemide dose weaned
6 (25/08/2020)	Temperature noted to be settling, haemodynamically stable
	Aspirin and therapeutic clexane started
7 (26/08/2020)	Repeat ECHO: ejection fraction: 50% methylprednisolone intravenous completed
8 (27/08/2020)	Switched to oral prednisone
9 (28/08/2020)	Stepped down to main medical ward
12 (31/08/2020)	Discharged

### Clinical findings

**Figure 1:**
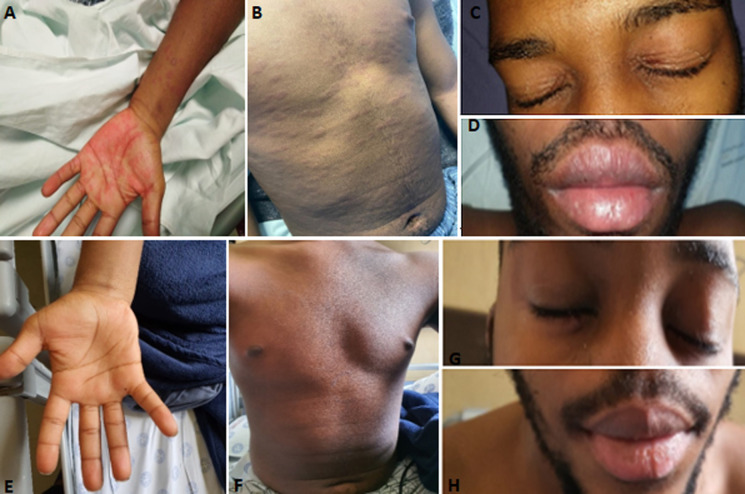
images of rashes on presentation and after treatment. Before: A) maculopapular, erythematous rash over palmar surface hand; B) papular rash over torso; C) desquamating rash over eyelids; D) mucositis of lips; after: E) resolution of rash over palms; F) torso; G) eyelids; H) lips

On clinical examination the patient was ill looking with a blood pressure of 103/44 mmHg, heart rate of 123 beats per minute and a temperature of 39.5°C. He was restless with no evidence of cognitive dysfunction. A generalized maculopapular rash was observed over the torso, upper back, limbs and palmar surface of the hands. The rash was non-tender, with erythematous desquamating lesions over the eyelids and bilateral conjunctivitis (Figure 1). The abdominal and respiratory examinations were normal with no jaundice, or lymphadenopathy. His cardiovascular examination revealed normal heart sounds with no added sounds, a sinus tachycardia, a normal volume pulse and warm, well perfused peripheries. He had no meningism and there was no arthritis.

### Diagnostic assessment

#### Diagnostic methods

Prior to arrival to CHBAH the patient had received three days of ceftriaxone and intravenous (IV) paracetamol with no clinical improvement. Blood investigations showed a rising C-reactive protein (CRP), procalcitonin (PCT), white cell count (WCC), ferritin, lactate dehydrogenase (LDH) and D-dimers (Table 2). Blood and urine cultures remained negative. The lumbar puncture was normal (including viral panel) with a normal chest radiograph and abdominal sonar. The first echocardiogram (ECHO) performed revealed a structurally normal heart with normal systolic function. Three nasopharyngeal swabs sent for SARS-CoV-2 polymerase chain reaction (PCR) were negative.

**Table 2:** laboratory findings through admission, HC-hydrocortisone, IVIG-Intravenous immunoglobulin, MP-methylprednisolone

	17/08/2020	Day 1	Day 2	Day 3	Day 4	Day 5	Day 6	Day 7
			HC		IVIG		MP	
**White cell count (3.92-10.40 x10^9^/L)**		10.8		16.77	16.96	13.28		10.71
**Haemoglobin (13.4-17.5 g/dL)**		13.5		11.1	10.8	10.1		8.8
**Neutrophil count (1.60-6.98 x109/L)**		10.4				13.7		
**Lymphocyte count (1.40-4.20 x10^9^/L)**		0.35				1.44		
**Creatinine (36-96 μmol/L)**	77			105	99			74
**ESR (0-10 mm/hr)**	54					119		
**CRP ((<10 mg/L)**	334		303		307			125
**PCT (<0.1 μg/L)**		17.83		19.97				2.71
**D-dimer (0.0-0.25 mg/L)**		0.87			2.46			1.63
**Direct bilirubin (0-3 μmol/L)**	36			8		7		
**Albumin (32-47 g/L)**	43			23		24		27
**ALT (5-30 U/L)**	36			35		102		89
**AST (0-39 U/L)**	35			78		113		82
**Ferritin (14-152 μg/L)**			1259		3764			
**LDH (100-190 U/L)**			356		543			302
**CK (22-198 U/L)**			245		307	316		
**Troponin T (<14 ng/L)**				842	797	216		

#### Diagnosis and differentials

An initial wide differential diagnosis of infections was considered: viral infections such as human immunodeficiency virus (HIV) seroconversion, acute infection with Epstein-Barr virus (EBV), herpes simplex virus (HSV) or cytomegalovirus (CMV); and bacterial infections caused by Group A streptococci, *Neisseria meningitidis*, other gram negative bacteria, rickettsia, leptospirosis or malaria. Non-infectious differential diagnoses were also considered: Kawasaki like disease (KD) as well as adult onset Still´s disease (AOSD) were considered in view of the rash, sore throat, elevated ferritin, CRP and erythrocyte sedimentation rate (ESR) in association with neutrophilia. Systemic lupus erythematosus (SLE) was considered on account of fever, rash and low complement (C3, C4) levels. An elevated temperature, rising CRP, PCT, LDH, D-Dimer and ferritin levels with deranged liver function tests (LFT), suggested a diagnosis of MIS-C.

#### Therapeutic intervention

**Figure 2:**
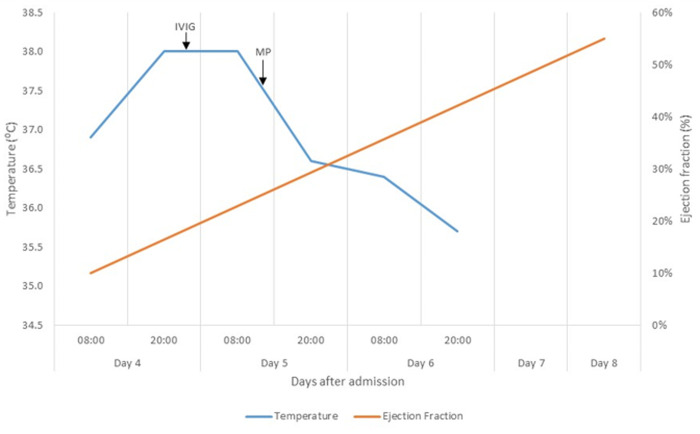
graph comparing changes in ejection fraction with temperature. Elevated Temperature shown to settle with methylprednisolone (MP) and intravenous immunoglobulin (IVIG)

On admission, the patient was tachycardic with a mean arterial pressure of 64mmHg, with no clinical or biochemical indicators of end organ hypoperfusion. Intravenous fluid boluses of crystalloid were administered with no significant response in haemodynamic parameters. Fluid boluses were ceased and urine output and serum lactate levels were monitored as markers of tissue hypoperfusion. Inotropes and vasopressors were not initiated as part of initial management. Doxycycline was added as empiric cover for rickettsial disease and ceftriaxone was continued on day two. Hydrocortisone was commenced on admission but discontinued on day three due to concern for infection. A transient resolution of the fever coincided with steroid initiation, and a subsequent elevation in temperature was noted when the steroids were interrupted (Figure 2). On day four of admission, in light of an audible gallop rhythm, persistently low mean arterial pressures and newly elevated cardiac troponin-T levels, a repeat ECHO was performed, revealing an ejection fraction (EF) of 10% (Figure 2) with global hypokinesia, four chamber dilation, moderate functional tricuspid and mitral regurgitation with a sliver of pericardial effusion. Intravenous immunoglobulin (IVIG) was started at a total dose of 120g (2g/kg) over 72 hours in conjunction with a dobutamine infusion and gentle diuresis to counter fluid overload.

An IV methylprednisolone pulse of 500mg daily for three days was initiated with the IVIG on day five of admission. Serological testing for SARS-CoV-2 was performed at the National Institute for Communicable Diseases (NICD) and confirmed the presence of immunoglobulin (IgG) antibodies against the SARS-CoV-2 trimeric spike protein with in-house enzyme-linked immunosorbent assay (ELISA) testing. A bone marrow aspirate and trephine (BMAT) revealed increased macrophage activity, but did not meet criteria for macrophage activation syndrome (MAS). Doxycycline and ceftriaxone were discontinued after four and seven days, respectively. Colchicine was added to cover for possible associated myopericarditis. Defervescence was noted on day six (Figure 2), accompanied by a downward trend in inflammatory markers, LDH and ferritin. Aspirin (300mg daily) and a therapeutic dose of enoxaparin (60mg twice daily) were added in view of thrombotic risk. The patient improved clinically with no further temperature spikes and repeat ECHO revealed an improved EF of 45% on day seven. Inotropic support was weaned off on day seven. Prednisone 30mg daily was initiated on day eight after completion of methylprednisolone pulse. The patient was monitored for a further two days, and showed clinical improvement, with no further temperature spikes and/ or hypotensive episodes. There was almost complete resolution of the initial rash. His WCC, CRP, PCT, LDH, D-dimers and ferritin were all on a downward trend and the LFT had normalized. The patient was discharged on day 12 on prednisone 20mg daily, aspirin 100mg daily, and colchicine 0,5mg twice daily (Table 1).

#### Follow up and outcomes

Mr. LN was reviewed 2 weeks later in the rheumatology outpatient clinic and was noted to have recovered fully with no residual symptoms. On examination there was complete resolution of the rash, with only few areas of residual hyperpigmentation. He had no tachycardia or signs of heart failure. A repeat ECHO showed an improvement in EF to 57%, without a pericardial effusion. The prednisone was weaned to 10mg daily and tapered down over the next four weeks. The treatment was well tolerated with no side effects noted. The patient returned to school and will be reviewed in the coming months.

#### Informed consent

Informed consent was obtained from the patient to use historical and clinical information as well as images avoiding identifiable features.

## Discussion

### Strengths and limitations

Our case study highlights a new disease phenomenon being increasingly recognized during the COVID-19 pandemic with clues to how it may present in our African population. This case report prompts further, larger studies of MIS-C in our setting. We have also included follow up findings and images illustrating the response to treatment. This was a single centre study and generalizations about this disease entity cannot be made.

### Clinical features

Similar to our patient, studies have noted a predominance of this condition in the African population with up to 52% of patients having had no associated comorbidities [5]. Patients with MIS-C present with features denoting a hyperinflammatory response secondary to a cytokine storm after infection [6]. Previous case series have reported fever, rash, conjunctivitis and gastrointestinal symptoms (abdominal pain, vomiting, diarrhoea) as predominant clinical features seen in MIS-C, as noted in our patient, compared to acute COVID-19 which may present with more upper respiratory tract symptoms [5,6]. Similar findings were seen in adult patients with MIS-A [4]. The presentation of MIS-C follows infection with SARS-CoV 2. Typical features may develop three to five weeks post infection [4,5]. A large proportion of cases were found to have positive serological testing as opposed to positive reverse transcription polymerase chain reaction (RT PCR) for SARS-CoV-2 from nasopharyngeal testing. This finding indicates that MIS-C may develop in the post infectious period during the hyperinflammatory phase (stage III) following SARS-CoV-2 infection [6]. Cardiac involvement is an important feature presenting as persistent tachycardia, arrhythmias, and hypotension and in some cases, cardiogenic shock [5].

### Investigations

Raised inflammatory markers are a hallmark of MIS-C including elevation in PCT, CRP, ferritin, and ESR, as seen in our patient. Interleukin 6 (IL-6) levels are also noted to be higher in this group [6]. Haematological investigation commonly reveals a neutrophilia and lymphocytopaenia which point toward more severe disease [5]. Elevated D-dimers are also observed. Our patient presented with troponin levels that were markedly raised, a common feature indicative of underlying myocardial dysfunction [5,6]. Prevalent findings on ECHO include low EF with depressed shortening fraction and pericardial effusion. A small proportion of patients present with an EF of < 10% with shock noted in up to 60% of these patients [5]. In some cases, as in our patient, the initial ECHO may be normal with myocardial dysfunction only developing days later [5]. Recovery was seen in a large proportion of patients following therapy [5]. Despite presenting with a history of significant abdominal pain our patient´s clinical (abdominal) examination and abdominal sonar were normal. Case series have shown ascites, intestinal inflammation and mesenteric adenopathy found on abdominal sonar, computed tomography scan and magnetic resonance imaging [5] of the abdomen.

### Antibodies

Studies have reported the onset of MIS-C occurring three to five weeks post SARS-CoV-2 infection with antibody testing playing a key role in diagnosis [5,6]. Seroconversion typically occurs three weeks post infection with with IgM concentration levels peaking 10-12 days after symptoms, followed by IgG levels after 12-14 days [7]. Our patient was found to have the presence of IgG antibodies indicating possible infection within the preceding weeks. Higher IgG titres have been documented to correlate with severe disease, indicating a role in prognostication [7].

### Diagnostic approach

Following a release by the CDC of the definition of MIS-C, guidelines have been formulated regarding assessment and management. According to local groups from the Western Cape. Features that should be noted on clinical examination include fever (>38°C), rashes, headaches, conjunctivitis, oedema, mucositis and lymphadenopathy on general examination. System examination may reveal meningism, confusion, generalized abdominal pain as well as hypotension or shock, if significant cardiac involvement is present. Baseline investigations include full blood count (FBC), D-Dimer, ferritin, PCT, LDH, LFTs, international normalized ratio (INR), triglycerides and blood cultures. Other helpful bedside investigations include urine dipstick and electrocardiogram (ECG). Serology for SARS-CoV-2 should be drawn if MIS-C is suspected. Troponin and pro brain natriuretic peptide (pro-BNP) are recommended if clinically indicated or if any new abnormalities are noted on ECG. Further tests include lumbar puncture, ECHO, as well as chest radiograph and/or abdominal ultrasound if indicated [8].

### Differential diagnosis

Important differential diagnoses considered in this patient included infectious causes such as viral, (including HIV seroconversion), and bacterial infections. The viral panel on serum and cerebrospinal fluid was however negative and no organisms were cultured on repeated blood cultures and specific serological testing. HIV seroconversion may present as acute retroviral syndrome within two to four weeks of exposure with findings common to MIS-C being fever, rash, diarrhoea and headache [9]. However, our patient tested HIV negative with a lower than detectable HIV load which is usually observed in early infection. Non-infectious entities such as KD or Kawasaki disease shock syndrome were considered, which like MIS-C, may present with predominant cardiac features such as ventricular dysfunction and coronary artery dilation, however the former does not usually present with abdominal symptoms and an elevated D-dimer and activated partial thromboplastin time as in the case of our patient and is usually found in a younger age group. Typical features of AOSD include spiking fevers, an evanescent rash and arthritis or arthralgia in the setting of hyperferritinaemia [10]. Despite presenting with a generalized maculopapular rash, our patient´s fever was persistent with no associated quotidian or double quotidian pattern, no arthritis or arthralgia, and no prominent lymphadenopathy or splenomegaly, thereby not fulfilling the Yamaguchi criteria for AOSD [10]. Features in keeping with SLE included fever and a rash with pericardial effusion and low complement levels. However, there was no history of arthralgia, rashes or other related symptoms prior to presentation and no arthritis, alopecia or other typical rashes. The anti-nuclear antibody was negative. The development of secondary haemophagocytic lymphohistiocytosis (HLH) or MAS was considered in view of elevated CRP, D-dimer, ferritin, deranged LFT and haemophagocytosis on bone marrow, however cytopaenias were not prominent with normal triglyceride level. Cardiac and gastrointestinal symptoms are not common features, as seen in our patient. The criteria for HLH were not met [6].

### Management

Management guidelines have been drawn from the treatment of KD and other associated rheumatological diseases. The aims of treatment are to abate systemic inflammation and prevent further organ dysfunction. Currently there remains no set algorithm regarding treatment, with IVIG and glucocorticoids recommended alone or in combination [6,8]. Studies have shown a positive response with combination treatment in those with cardiac involvement [8]. This was evidenced in our patient, who demonstrated significant defervescence and overall clinical improvement with recovery in cardiac function after the onset of IV methylprednisolone followed by IVIG infusion. Patients refractory to IVIG and steroids may require escalation of therapy to anakinra (interleukin-1 type I receptor antagonist) or tocilizumab (IL-6 inhibitor) [6,8]. Tocilizumab is preferred in those with HLH [6]. Due to the risk of thrombosis our patient was placed on low dose aspirin. Prophylactic doses of low molecular weight heparin have been suggested in those with significantly depressed EF which was introduced in our patient and discontinued later after recovery of left ventricular function.

## Conclusion

Our patient´s case along with others, illustrates the importance of early recognition and diagnosis of MIS-C due to the rapid clinical deterioration associated with the hyperinflammatory state. This condition is now increasingly seen in the adult and adolescent population highlighting the need for heightened awareness amongst those treating adults. Careful consideration is required due to the nonspecific nature of symptoms, clinical features and the wide differential which includes infection in our sub-Saharan African setting. Importantly, lack of access to serological testing for SARS-CoV-2 should not delay diagnosis and management of MIS-C, as early treatment is crucial in preventing further organ damage. This case also highlights the importance of collaboration between adult medical and paediatric specialists. Further studies are required in our setting to monitor the outcome and long-term sequelae in these patients with MIS-C.
